# Comparing the Effect of Two Educational Programs on the Quality of Life of Hemodialysis Patients in Iran

**DOI:** 10.5812/ircmj.19368

**Published:** 2014-08-05

**Authors:** Shahram Baraz, Kourosh Zarea, Bahman Dashtbozorgi

**Affiliations:** 1 Chronic Diseases Care Research Center, Nursing and Midwifery School, Ahvaz Jundishapur University of Medical Sciences, Ahvaz, IR Iran

**Keywords:** Hemodialysis, Quality of Life, Patient Education, Audiovisual Demonstration, Iran

## Abstract

**Background:**

Various researchers have shown that the health level, performance status, and quality of life (QOL) are often less than expected especially in hemodialysis (HD) patients.

**Objectives:**

This study aimed to determine the effect of the two methods of educational programs on health- related QOL (HRQOL) in Iranian HD patients.

**Patients and Methods:**

In this quasi-experimental, pretest-posttest interventional study, we employed each subject as his/her own control. The study was conducted at the dialysis units in three major general hospitals affiliated with Ahvaz Jundishapur University of Medical Sciences. A total of 90 HD patients were randomly allocated to two 45-patient groups of oral and video education programs, respectively. The educational programs included dietary and fluid regimens, the care of fistula and skin, and stress management. HRQOL was assessed in both groups using a Farsi version of the Short Form Health Survey (SF-36) before and after the educational programs. Repeated measures analysis of variance and ANOVA were used for data analysis through SPSS.

**Results:**

SF-36 domains of physical functioning (P < 0.021), role physical (P < 0.031), social functioning (P < 0.001) and mental health (P < 0.001) were significantly increased in both oral and vide education groups after the interventions. There was no difference in the effectiveness of the two educational programs.

**Conclusions:**

Appropriate interventions may potentially lead to improvement in the HRQOL of these patients. Therefore, video education as an effective, inexpensive, simple, and attractive method is recommended for HD patients.

## 1. Background

The prevalence rate of end-stage renal disease (ESRD) has increased by 8% from 2007 to 2012 (1). In 2008, over 16600 patients with ESRD were under treatment with maintenance hemodialysis (HD) in 355 dialysis units in Iran (2). Figures reported by the Ministry of Health indicate a 20% increase in the number of these patients; in Tehran alone, 00 patients are monthly added to the list (3). A broad range of factors including diet limitations, clinical manifestations of the disease, adverse effects of therapy, and changes in the lifestyle as well as social life affect the quality of life (QOL) and sense of socio-mental well-being in the patients undergoing HD (4-6). This raises the risk of morbidity and mortality in patients and imposes a heavy burden on the healthcare system (7). In Iran, the heavy costs of the disease afflict the family economy in the long term, while healthcare strategies fail to support these patients sufficiently (6). Most studies indicate that an improvement in health-related QOL (HRQL) would alleviate the complications of the disease or at least render them more tolerable (8). Improving the QOL constitutes a major goal in the treatment of chronically ill patients (9). Considering the chronic and debilitating nature of the ESRD, the patients’ need for long-term dialysis, and the effect of disease and therapy on their QOL, an educational program would be vital to address the patients’ QOL. It is crucial to involve the patients actively in the treatment program in order to efficiently control the disease complications and improve their QOL. It requires raising the awareness of patients and to achieve this goal, education serves as the most appropriate tool (9, 10). In a treatment team, nurses have the most extensive contact with the patients. Consequently, education is an essential component of their responsibilities as emphasized by standards of clinical practice developed by the American Nurses Association. The performed educational program by nurses will improve the patients’ awareness about health issues and reduce the detrimental effect of the disease on their QOL (9). Nurses are in direct charge of HD patients, educating the patients and their families, and encouraging the patients to care for themselves (9, 11). Oral education ins one of the most powerful modalities of education that provide the patient with an opportunity for active learning in real conditions while presenting the appropriate patterns, which are optimized for the personal characteristics of the trainee. One drawback of this method, however, is the posed difficulty by education during the dialysis and gathering the patients between the dialysis sessions. Therefore, it is crucial to discover new modalities that circumvent these challenges. The advances in communication technology have provided excellent and diverse modalities of communication such as video education. The advantages of this type of education include the possibility of saving and resuming information, convenience of use, and low cost. Nevertheless, it lacks the benefit of maintenance of an active contact by a present trainer, which undoubtedly contributes to the objectives of education. The recent breakthroughs in educational movies have made it possible to try and minimize this disadvantage (5, 12). Numerous studies conducted in various countries indicate that patients undergoing HD have lower QOL in comparison to the healthy population (13, 14). Therefore, these patients require special and persistent education in order to cope with their physical and mental challenges (15). Many studies have been conducted throughout the world to address the issue of education, which have targeted at improving the HRQOL in HD patients; these studies have mostly used oral or monitoring modalities (9, 16, 17).

## 2. Objectives

We could not find any published literature that had used video education for HD patients or had compared it with other modalities in Iranian population. Therefore, we conducted the present study to compare the effect of oral and video education on the QOL of the patients undergoing maintenance HD in Ahvaz, Iran.

## 3. Patients and Methods

### 3.1. Design

In this quasi-experimental, pretest-posttest interventional study, we considered each subject as his/her own control. The study was conducted in the dialysis units in three major general hospitals included Imam Khomeini Hospital, Golestan Hospital, and Razi Hospital affiliated with the Ahvaz Jundishapur University of Medical Sciences.

### 3.2. Sample Participants

**Figure 1.**
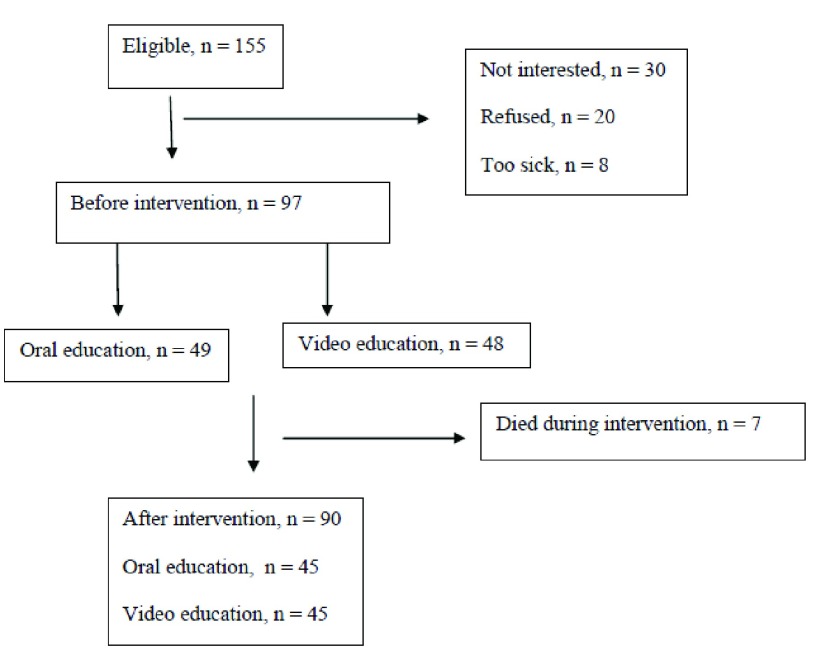
Recruitment and Data Collection Flow Chart

The data were collected from August 2013 to December 2013 in three major general hospitals affiliated with the Ahvaz Jundishapur University of Medical Sciences, Ahvaz, Iran. These hospitals were governmental and referral. The criteria for selecting the participants were a minimum age of 18 years, being under HD for at least six months, three times a week HD, each time for four hours under maintenance HD treatment, living at home, no participation in other training programs in this area before formal training and during the study, and reluctancy to participate in the study. The equation of Cochran was used to yield a representative sample for proportions. The sample size was calculated at 89. Expected power was estimated at about 0.8. On the other hand, all 155 HD patients at the three HD centers were required to take part in this study. Finally, 90 patients were recruited (Figure 1). Patients were selected based on the inclusion criteria and were randomly allocated into two groups. Random allocation was performed by using the random computer-generated numbers. A suitable table in a statistics textbook was used. The numbers were taken as random digits from zero to 99. For equal allocation to two groups, we took odd and even numbers for the oral and video education groups, respectively.

### 3.3. Data Collection

For measuring QOL, the Iranian version of the Short Form Health Survey (SF-36) was applied. This tool has been interpreted and validated for use in HRQOL assessment in Iran (18). It consists of eight subscales: physical functioning (PF), role limitations owing to physical health problems, bodily pain, social performance, general mental health, role limitations owing to emotional health problems, energy and weariness, and general health perception. The questionnaire consisted of eight dimensions; each scored from zero to 100. Questions with three choices were scored zero, 50, and 100, those with five choices were scored zero, 25, 50, 75, and 100, and those with six choices were scored zero, 20, 40, 60, 80, and 100, with higher scores depicting better function (4). The alpha coefficient of eight subscales obtained in the present study was > 0.85. The required data were collected within the first two hours after the initiation of HD in order to ensure that the subjects were not experiencing any dialysis-related discomfort.

### 3.4. Educational Program

Initially, the educational needs were defined with the help of patients and nurses. Then we devised an educational program with the assistance of nephrologists and dieticians. The program aimed to enable HD patients to care for themselves in the domains of diet, fluid intake, vascular route, skin care, and stress management. Therefore, two educational programs were designed and performed. The first program was conducted through holding group sessions and the educational contents were presented by oral presentation. In the second program, the same educational contents were presented through showing a video film for each patient. The patients were randomly allocated into two groups. In the first group (n = 45), the patients attended in the group education sessions, while the patients in the second group were considered for video education. A classroom in the HD center was considered for group education sessions. The patients in the first group attended in the educational sessions in the class on the days after their HD sessions (on Monday, Wednesday, and Saturday). Totally, two educational sessions were held. The duration of each session did not exceed more than 45 minutes. The principal investigator, who was a renal-expert nurse, performed the educational program. A teaching booklet was prepared by the researches and was given to each patient at the end of the group sessions. The booklet was a patient’s guide to control dietary regimen. Patients in the second group (video education) watched an educational film on a video disc during two consecutive dialysis sessions in a week. First, the patient was allowed to go to HD and after ensuring that the patient is stable and ready (usually following one to two hours after initiation of HD), he/she was invited to watch the 45-minute film. The educational contents of both programs were similar and covered necessary information about the ESRD and dietary management for HD, particularly fluid restrictions and identification of restricted/allowed foods, as well as skin care and stress management. Data were collected at pretest and six months following the educational program.

### 3.5. Ethical Considerations

Ethics Committee of the Ahvaz Jundishapur University of Medical Sciences, Ahvaz, Iran, approved the study with code ETH-733 (January 2013). The patients were sufficiently notified about the study method and objectives and each one signed a written informed consent.

### 3.6. Statistical Analysis

We used mean, standard deviation, and Chi square test to analyze the demographic data on SPSS (version 16, SPSS Inc., Chicago, IL, USA). Since this study consisted of two types of education (inter-group factor) and two evaluation points (intragroup factor), ANOVA with repeated measures was used for analysis. It should be noted that the study data distribution was normal. Finally, the overall mean for both educational programs was calculated to determine which one has improved the dimensions of QOL. Moreover, to find out whether or not the subjects’ QOL had differed over time, the overall scores were compared before and after the educational programs. To reveal any interaction between the educational programs and time, the means for each group at each time (preintervention or postintervention) were analyzed. In order to compare the overall mean before and after education, the ANOVA test was used with Bonferroni correction of P value < 0.005.

## 4. Results

### 4.1. Demographic Characteristics

The demographic and basic characteristics of the participants are shown in Table 1. Chi-square and an independent-samples t-test did not reveal any significant difference between the two groups at baseline in terms of the demographic variables (Table 1).

**Table 1.** Demographic Characteristics of the Study Participants^a^

Variables	Education Program		P Value
	Oral (n = 45)	Video (n = 45)	
**Sex**			0.9
Male	51.1	46.6	
Female	48.9	53.4.4	
**Education**			0.53
Elementary School	15.5	22.25	
Secondary or Higher	35.5	22.25	
College	49	55.5	
**Marital Status**			0.45
Single	35.5	42.2	
Married	64.5	57.8	
**Occupation**			0.35
Unemployed	77.8	68.9	
Employed	22.2	31.1	
**Mean Age, y**	35.87 ± 10.13	33.83 ± 8.89	0.34
**Mean Duration of Hemodialysis, y**	4.32 ± 2.54	4.9 ± 2.52	0.93

^a^Data are presented as are mean ± SD or No. (%).

### 4.2. Quality of Life

As shown in Table 2, in the oral education group, educational intervention was associated with a statistically significant increase in QOL dimensions (physical functioning, physical role, social function, vital force and energy, mental health, and overall health); however, regarding the general health, emotional role, and bodily pain dimensions, the differences were insignificant. Similarly, in the video group, changes in the HRQOL dimensions (physical functioning, physical role, emotional role, social function, mental health, and overall health) showed significant differences after the educational program. Despite of relative changes in general health, vital force and energy, and physical pain dimension, the difference was not significant. Table 2 compares the overall scores before and after the educational program in the two groups in order to determine whether or not the subjects differed over time. The statistical analysis showed that the overall mean of physical functioning, physical role, social function, vital force and energy, mental health, and overall health increased after the educational program, which meant changes in individuals over time.

**Table 2.** Mean Score of Quality of Life and Overall Means Before and After Educational Program in Hemodialysis Patients^a,b,c^

Score of Quality of Life	Education		
	Before	After	P Value
**General Health Perception**			
Oral	40.62 ± 17.87	41.01 ± 16.87	0.82
Video	48.38 ± 22.99	48.38 ± 18.18	1
Overall mean	44.44 ± 21.15	44.64 ± 17.72	0.94
**Physical Functioning**			
Oral	63.9 ± 17.3	70.15 ± 13.4	0 < 001^d^
Video	60.32 ± 20.89	68.63 ± 22.82	0.018^d^
Overall Mean	62.14 ± 19.08	69.4 ± 18.15	0.021^d^
**Role Physical**			
Oral	43.22 ± 17.2	50.51 ± 18.9	0.003^d^
Video	50 ± 20.1	60.48 ± 22.14	0.005^d^
Overall Mean	46.56 ± 35.81	54.62 ± 33.41	0.031^d^
**Role Emotional**			
Oral	44.25 ± 16.9	44.76 ± 19.7	0.9
Video	41.91 ± 19.35	50.53 ± 21.92	0.018^d^
Overall Mean	43.08 ± 16.8	47.64 ± 23.84	0.26
**Social Functioning**			
Oral	55.46 ± 16.47	64.06 ± 19.24	0.003^d^
Video	60.08 ± 24.66	67.74 ± 20.09	0.035^d^
Overall Mean	57.53 ± 20.87	65.87 ± 19.59	0 < 001^d^
**Pain**			
Oral	57.5 ± 30.9	55.45 ± 29.14	0.39
Video	59.03 ± 32.38	53.22 ± 32.34	0.32
Overall Mean	58.25 ± 31.39	54.35 ± 30.53	0.21
**Energy and Fatigue**			
Oral	46.31 ± 19.97	56.1 ± 20.6	0.005^d^
Video	48.73 ± 21.33	48.95 ± 15	0.94
Overall Mean	47.48 ± 20.51	52.45 ± 18.46	0.034^d^
**Mental Health**			
Oral	46.28 ± 27.29	55.07 ± 27.9	0.004^d^
Video	43.55 ± 12.8	49.84 ± 18.84	0.01
Overall Mean	44.94 ± 21.25	52.3 ± 20.39	0 < 001^d^
**Overall Health**			
Oral	49.60 ± 15.66	54.47 ± 13.71	0 < 001^d^
Video	52.25 ± 19.04	56.17 ± 16.29	0.002^d^
Overall Mean	50.90 ± 17.31	55.31 ± 14.93	0.003^d^

^a^Data are presented as means ± SD.

^b^In order to compare overall mean before and after education, the ANOVA test was used with Bonferroni correction of P value < 0.005.

^c^In each group, 45 patients were allocated randomly.

^d^The difference is statistically significant.

### 4.3. Comparison of Two Educational Programs

To determine whether the two educational programs resulted in different scores, the overall means of the two study groups were compared (Table 3) and no significant difference was observed between the overall means of the QOL scores in the two groups.

**Table 3.** Overall Means of Short Form Health Survey Scores for the Two Teaching Groups^a^

Score of Quality of Life	Overall Mean of Education Programs		P Value^b^
	Oral (n = 45)	Video (n = 45)	
**General Health**	40.52 ± 17.92	44.38 ± 20.55	0.21
**Physical Function**	67.03 ± 15.67	64.47 ± 22.10	0.47
**Role Physical**	46.72 ± 32.62	54.43 ±37.19	0.24
**Role Emotional**	44.87 ± 33.13	46.22 ± 38.34	0.83
**Pain**	56.52 ± 30.21	56.12 ± 32.23	0.94
**Social Function**	59.67 ± 18.44	62.91 ± 22.64	0.26
**Energy and Fatigue**	50.88 ± 21.13	48.61 ± 18.23	0.54
**Mental Health **	50.7 ± 17.78	46.49 ± 24.18	0.24
**Overall Health**	52.01 ± 14.93	53.98 ± 17.62	0.509

^a^Data are presented as means ± SD.

^b^In order to compare the overall mean before and after education, ANOVA test was used with Bonferroni correction of P value < 0.005. There was no significant difference between the overall means of quality of life scores in the two educational programs.

## 5. Discussion

The results of this study showed that oral and video education could affect the health related QOL dimensions in the patients and improve their QOL. The results also showed that there was no significant difference between the effectiveness of the abovementioned educational programs. The importance of educational programs and their effect on HRQOL for HD patients have been studied by several studies. Wong et al. showed that the effective management model of chronic kidney disease in which the patient took the self-care role, could result in better acceptance of diet and treatment regimens by patients and hence, improved HRQOL and life satisfaction in HD patients (19). A study by Thomas et al. in India showed that education might influence the dimensions of QOL in HD patients three to six months after the educational intervention (17). In line with our study, a study in Taiwan showed that the patients' HRQOL and self-care self-efficacy were increased dramatically after the educational program (9). Most patients with chronic renal failure who are unaware of the complications as well as incurable nature of their disease are forced to undergo routine dialysis. Patients often deny their condition and do not adhere to the treatment regimens, resulting in increased symptoms and reduced health related QOL. The low level of self-care and reduced HRQOL in HD patients in our study indicated that the patients really did experience low levels of self-care. This is comparable with the findings of other studies (9). Lev and Owen expressed that lower self-care and inability to perform self-care shows low affinity of the patients to perform self-care behaviors. These patients need educational programs that could dramatically increase their ability to employ self-care behaviors (20). The results of this study are in agreement with findings from other studies that used SF-36 to assess HRQOL for HD patients (21, 22). HRQOL is an important index for assessing the outcome of medical treatment. As self-care is the main predictor of HRQOL in patients with ESRD, educational programs to promote self-care and improve the patients' HRQOL are essential. The findings of this study indicated that the two educational programs were not significantly different in terms of effect on the QOL of HD patients. Moreover, we found no statistically significant interaction between the main variables (oral and video education) and time (before and after education). Few studies have suggested that video education may improve self-care behaviors in the patients with chronic conditions (23, 24). Nonetheless, all of these studies had dealt with video education only and had not compared it to other educational modalities (e.g. audio or oral education). On the other hand, as our search in the literature did not yield a study that had compared oral with video education in terms of QOL in HD patients, we could not compare our results with those of other studies. Although some suggest that video education entails certain benefits for the patients and their families (25), our findings fail to indicate any significant difference between the two educational modalities. In summary, our findings demonstrated that education, either oral or video, improved the QOL for HD patients. Increasing the knowledge and awareness of HD patients and improving their QOL must constitute a cornerstone of therapy and an integral part of nursing responsibilities. Nurses should educate the patients about self-care behaviors and remind them of the dangerous complications of abandoning these behaviors.

### 5.1. Limitations

The present study was conducted over a period of six months to compare the effect of two educational programs on the dimensions of QOL in HD patients. Thus, one of its limitations was the short-term follow-up of patients. Further studies are required to investigate whether these early beneficial effects persist over longer durations or not. Another limitation to the present study was the relatively small number of patients, which necessitate the conduction of further studies with larger sample sizes and longer follow-up period. Yet another limitation was the lack of a control group, which must be considered in the future studies. The final limitation was that the present study only addressed HD patients; therefore, its findings may not be applicable to other groups of patients.
